# Regional inequalities and temporal trends in maternal disorders across sub-Saharan Africa, 1990–2023: a systematic analysis of the global burden of disease 2023 data

**DOI:** 10.1186/s13690-026-01979-z

**Published:** 2026-06-04

**Authors:** Yusuf Hared Abdi, Yakub Burhan Abdullahi, Mohamed Sharif Abdi, Naima Ibrahim Ahmed, Sharmake Gaiye Bashir, Ahmed Abdiaziz Alasow, Olalekan John Okesanya, Yusuff Adebayo Adebisi, Don Eliseo III Lucero-Prisno

**Affiliations:** 1 Faculty of medicine and Health science, Hormuud University, Mogadishu, Somalia; 2 Department of Medical Laboratory Science, Neuropsychiatric Hospital, Aro, Abeokuta, Nigeria; 3 Faculty of Medicine, Department of Public Health and Maritime Transport, University of Thessaly, Volos, Greece; 4 College of Social Sciences, University of Glasgow, 40 Bute Gardens, Glasgow, G12 8RT UK; 5 Department of Global Health and Development, Faculty of Public Health and Policy, London School of Hygiene and Tropical Medicine, London, UK; 6 Office for Research, Extension and Innovations, Bukidnon State University, Malaybalay City, Bukidnon, Philippines; 7 Research Office, Palompon Institute of Technology, Palompon, Leyte, Philippines

**Keywords:** Maternal disorders, Sub-Saharan Africa, Regional inequalities, Temporal trends, Global Burden of Disease, Maternal mortality, Disability-adjusted life years, Maternal hemorrhage, Hypertensive disorders

## Abstract

**Background:**

Maternal disorders remain a critical public health challenge in sub-Saharan Africa, accounting for disproportionate maternal mortality despite global progress. This study examined regional inequalities and temporal trends in maternal disorders across sub-Saharan Africa from 1990 to 2023 using the Global Burden of Disease 2023 data.

**Methods:**

Age-standardized disability-adjusted life years and death rates per 100,000 population were extracted for 11 maternal disorder causes across 46 sub-Saharan African countries classified into four subregions (Western, Eastern, Central, and Southern Africa). Cause of Death Ensemble modeling and DisMod-MR 2.1 were used to generate mortality and morbidity estimates. Temporal trends were assessed using percentage change calculations, and geographic distributions were mapped using choropleth visualizations. Subregional inequalities were quantified by comparative analyses of absolute and relative disparities.

**Results:**

In 2023, maternal disorders accounted for 1,601.9 disability-adjusted life years and 25.8 deaths per 100,000 population region-wide, with substantial subregional variation. Central Africa exhibited the highest burden (2,592.6 disability-adjusted life years; 42.7 deaths per 100,000), followed by Western Africa (1,889.6; 30.9), Eastern Africa (1,204.4; 18.7), and Southern Africa (586.3; 9.4). Maternal hemorrhage, sepsis, and hypertensive disorders were the leading causes. From 1990 to 2023, the overall burden declined by 60%, driven primarily by Eastern Africa’s 76.6% reduction in disability-adjusted life years. Central and Western Africa achieved moderate declines (42.8% and 50.2%, respectively), whereas Southern Africa experienced increases in specific causes, including hypertensive disorders (33.5%) and HIV-aggravated maternal deaths (241.5%).

**Conclusion:**

Despite remarkable progress, profound regional inequalities persist in the burden of maternal disorders across sub-Saharan Africa. Achieving Sustainable Development Goal 3.1 requires strengthened health systems, universal access to quality obstetric care, expanded reproductive health services, and targeted interventions that address subregion-specific maternal health challenges.

**Supplementary Information:**

The online version contains supplementary material available at 10.1186/s13690-026-01979-z.

**Table:** 

Text box 1. Contributions to the literature
• This study provides the first comprehensive subregional analysis of maternal disorders across all four sub-Saharan African regions using GBD 2023 data, covering 33 years and 46 countries.
• It highlights substantial and persistent inequalities, showing where progress is slow and where targeted interventions are urgently needed.
• By disaggregating the burden across 11 specific maternal disorder causes, the study offers granular evidence to guide priority setting for maternal health programs.
• The findings contextualize maternal disorder trends within global commitments such as SDG 3.1, supporting policymakers in designing region-specific strategies.

## Introduction

Maternal disorders remain among the most critical public health challenges confronting women of reproductive age globally, representing a constellation of preventable, yet life-threatening conditions. These encompass maternal hemorrhage, sepsis and other maternal infections, hypertensive disorders, obstructed labor and uterine rupture, abortion and miscarriage complications, ectopic pregnancy, and indirect maternal causes [1–3]. Within the Global Burden of Disease (GBD) framework, these conditions are systematically categorized to enable comprehensive epidemiological surveillance and evidence-based policy formulation in diverse geographical contexts [1, 2]. Despite substantial global progress in reducing maternal mortality over the past three decades, with age-standardized mortality rates declining by approximately 3.6% annually between 1990 and 2021, maternal disorders continue to impose a disproportionate burden on death and disability in women worldwide [2]. The internat ional community has codified this challenge within Sustainable Development Goal 3.1, which establishes an ambitious target of reducing the global maternal mortality ratio to less than 70 deaths per 100,000 live births by 2030 [1, 4, 5]. However, achieving this target remains profoundly uneven across regions, with substantial disparities in both progress and outcome [1, 2].

Sub-Saharan Africa (SSA) bears the most severe burden of maternal mortality globally, accounting for nearly two-thirds of all maternal deaths despite representing only a fraction of the world’s population [6–8]. The region’s maternal mortality ratio remains persistently elevated, with an estimated 536 deaths per 100,000 live births, more than twice the global average, underscoring the urgent need for targeted interventions and sustained investment in maternal health systems [9]. Moreover, the rate of decline in maternal mortality has been considerably slower in SSA than in other global regions, threatening the achievement of SDG 3.1 targets across the continent [2, 9, 10]. Critically, this aggregate regional burden masks profound heterogeneity in maternal health outcomes across the four sub-regions of SSA [10, 11]. Ethiopia and Rwanda have achieved remarkable declines in maternal mortality through systematic health system strengthening, expanded access to skilled birth attendance, and sustained political commitment to maternal health priorities [7, 12–14]. Conversely, nations, including Nigeria and South Africa, have experienced stagnation or even reversals in maternal mortality trends, driven by complex interactions between health system fragility, persistent socioeconomic disparities, armed conflicts, and inadequate access to emergency obstetric care [7, 11, 15]. These divergent trajectories highlight the critical importance of disaggregated analyses that illuminate within-region inequalities and country-specific patterns, which remain inadequately characterized in the existing literature [7, 11, 16].

Current evidence on maternal disorders in SSA suffers from significant analytical and methodological limitations that constrain policy-relevant decision-making [7, 16]. Previous national studies have often been fragmented, restricted to single countries or short time periods, and limited in their capacity to capture long-term temporal trends or to enable systematic cross-national comparisons [7, 16]. Furthermore, comprehensive examinations of subregional inequalities across Western, Eastern, Central, and Southern Africa using standardized metrics and consistent methodological frameworks remain scarce [7, 10, 11]. Most existing analyses have focused on aggregate maternal mortality without systematically disaggregating the burden by specific underlying causes such as hemorrhage, hypertensive disorders, or sepsis, thereby limiting the granularity of evidence available to inform targeted interventions [7]. The release of the GBD 2023 estimates presents a unique opportunity to address these critical gaps by providing harmonized, comparable data spanning three decades across all SSA nations and subregions [1, 2]. However, to date, no study has leveraged this comprehensive data to conduct a systematic comparative analysis of cause-specific maternal disorder patterns and their temporal evolution across sub-Saharan Africa’s diverse epidemiological and health system contexts.

The objective of this study was to examine regional inequalities and temporal trends in maternal disorders across SSA from 1990 to 2023 using data from the GBD 2023. This investigation represents the first comprehensive SSA-wide comparison encompassing 11 distinct maternal disorder causes systematically analyzed across four subregions, Western, Eastern, Central, and Southern Africa, over a 33-year period. By quantifying cause-specific burden, identifying divergent temporal trajectories, and characterizing persistent inequalities, this analysis provides critically needed policy-relevant evidence for tracking progress toward SDG 3.1 and universal health coverage commitments in maternal health [1, 15, 17]. The findings will enable policymakers, program managers, and global health stakeholders to identify priority areas for intervention, allocate resources more effectively, and design context-specific strategies that address the unique maternal health challenges confronting different SSA sub-regions [7, 11]. Ultimately, this study contributes to advancing the evidence base necessary to accelerate reductions in preventable maternal deaths and ensure that all women across SSA can access the quality of maternal healthcare services essential for their survival and well-being [1, 7, 15].

## Methods

### Study design and data sources

This analysis utilized data from the Global Burden of Disease Study 2023, conducted by the Institute for Health Metrics and Evaluation (IHME) at the University of Washington [18]. The GBD 2023 study represents the most comprehensive global effort to systematically quantify health loss from diseases, injuries, and risk factors across 204 countries and territories. We extracted estimates for maternal disorders spanning the period 1990 to 2023 for all 46 countries in SSA, which were classified into four subregions (Western, Eastern, Central, and Southern Africa) according to the United Nations geoscheme classification system. Data on maternal disorders were obtained through the GBD 2023 Results Tool, a publicly accessible database that provides age-standardized estimates with uncertainty intervals for deaths, disability-adjusted life years, years of life lost, and years lived with disability. All GBD data are generated through systematic identification, cataloguing, and processing of multiple data sources, including vital registration systems, verbal autopsy studies, surveillance systems, population-based surveys, censuses, and published literature. The GBD 2023 study incorporated 86,249 distinct data sources globally, including 19,354 sources reporting deaths and 31,499 sources reporting incidence data [18]. Each data source undergoes rigorous quality assessment and standardization procedures to ensure comparability across countries, time periods, and causes of death [18].

### Case definitions and cause classification

Maternal disorders were defined according to the GBD cause hierarchy, which categorizes maternal deaths as any death occurring in women while pregnant or within one year of termination of pregnancy from causes related to or aggravated by the pregnancy or its management, excluding accidental or incidental causes [18]. The analysis encompassed eleven specific maternal disorder causes: maternal hemorrhage, maternal sepsis and other maternal infections, maternal hypertensive disorders, maternal obstructed labor and uterine rupture, maternal abortion and miscarriage complications, ectopic pregnancy, other direct maternal disorders, indirect maternal deaths, late maternal deaths, maternal deaths aggravated by HIV/AIDS, and an aggregate category for overall maternal disorders. These cause-specific categories were defined using International Classification of Diseases codes (ICD-9 codes 630–676 and ICD-10 codes O00-O99) and aligned with previous GBD iterations to ensure temporal comparability across the 33-year study period [19].

### Statistical modeling and estimation methods

Cause-specific mortality estimates were generated using the Cause of Death Ensemble model, a sophisticated analytical framework developed specifically for the GBD study that systematically evaluates hundreds of plausible statistical models and combines them into an ensemble with optimal out-of-sample predictive performance [20]. The CODEm approach explores four distinct model classes: mixed-effects linear regression models of the natural logarithm of cause-specific death rates, mixed-effects linear regression models of the logit-transformed cause fraction, spatiotemporal Gaussian process regression models of the natural logarithm of cause-specific death rates, and spatiotemporal Gaussian process regression models of the logit-transformed cause fraction [20, 21]. Each model class incorporates relevant covariates identified through systematic covariate selection algorithms that assess theoretical plausibility and empirical associations with maternal mortality, including indicators of healthcare access, sociodemographic development, skilled birth attendance coverage, and fertility rates [21]. All candidate models undergo rigorous out-of-sample predictive validity testing using multiple holdout patterns, and the best-performing models are weighted proportionally according to their predictive accuracy to generate final ensemble estimates [21]. This approach has been shown to outperform any single component model in achieving lower root mean square error, higher frequency of predicting correct temporal trends, and improved coverage of prediction intervals [21].

For causes where sufficient epidemiological data on incidence, prevalence, remission, and case fatality existed, the GBD study employed DisMod-MR 2.1, a Bayesian meta-regression tool designed to enforce internal consistency between epidemiological parameters through compartmental disease modeling [20, 21]. DisMod-MR integrates data from multiple sources while accounting for known methodological and ecological determinants through the incorporation of study-level and country-level covariates [21]. The model applies spatial and temporal smoothing to borrow strength across neighboring locations and adjacent time periods, enabling estimation in data-sparse settings while preserving observed trends in data-rich locations. Disability-adjusted life years were calculated as the sum of years of life lost due to premature mortality and years lived with disability, following standard GBD methods. Years of life lost were computed by multiplying the number of deaths in each age-sex-location-year group by the residual life expectancy from standardized GBD reference life tables. Years lived with disability were estimated by multiplying the prevalence of each sequela by its associated disability weight, with adjustments for comorbidity using simulation methods to avoid double-counting of disability across multiple concurrent conditions.

### Age standardization and uncertainty quantification

All mortality and DALY rates were age-standardized to the GBD standard population to enable valid comparisons across countries and time periods with differing age structures [18]. Age standardization employed direct standardization methods, applying age-specific rates observed in each location-year to the standard population age distribution and summing across age groups to produce age-standardized rates per 100,000 population [18]. Uncertainty in all estimates was propagated through the entire estimation process using a simulation framework that generated 1,000 posterior draws for each quantity of interest. For CODEm, draws were created from the ensemble model with the number of draws contributed by each component model proportional to its ensemble weight. For DisMod-MR estimates, draws were generated using Markov Chain Monte Carlo sampling from the posterior distribution of epidemiological parameters. Final point estimates represent the mean of the 1,000 draws, and 95% uncertainty intervals were defined as the 2.5th and 97.5th percentiles of the draw distribution, capturing both sampling uncertainty in input data and model specification uncertainty [21].

### Temporal trend analysis

Temporal trends were assessed by calculating the Average Annual Percent Change (AAPC) using log-linear regression models of the form ln(rate) = α + β(year) for the entire period 1990–2023. The AAPC was derived as (e^β − 1) * 100, and 95% confidence intervals (CIs) were computed based on the standard error of the slope coefficient to quantify statistical uncertainty in the trends.

### Geographic mapping and subregional comparisons

Geographic distributions of maternal disorder mortality across SSA were visualized using choropleth maps constructed with shape files obtained from the Global Administrative Areas database. Country-level age-standardized death rates were mapped for both 1990 and 2023 to illustrate spatial patterns and temporal changes in the geographic distribution of maternal mortality. Subregional inequalities were quantified by comparing 2023 age-standardized rates across the four SSA subregions and calculating both absolute differences (arithmetic differences in rates between subregions) and relative differences (ratios of rates between subregions). The subregion with the highest burden was identified for each maternal disorder cause, and disparities were characterized by comparing this maximum burden to the subregional minimum, providing measures of within-region inequality.

To assess whether subregional aggregations masked heterogeneity or were driven by high-population countries, we performed a country-level sensitivity analysis by ranking all 46 countries according to their AAPC and examining the distribution of countries within each subregion. We compared the unweighted median AAPC across countries within each subregion to the weighted (population-based) subregional averages to identify potential contribution of high-population nations.

### Data management and analytical software

All statistical analyses, data processing, and visualization were conducted using R Studio version 4.5.1. Key R packages employed included “data.table” for efficient manipulation of large epidemiological datasets, “ggplot2” for construction of publication-quality graphics and temporal trend visualizations, “sf” for spatial data handling and geographic mapping, and custom packages developed by IHME for interfacing with the GBD Results Tool application programming interface. Sensitivity analyses were performed to assess the robustness of findings to alternative methodological choices, including examination of the impact of different age standardization reference populations and alternative approaches to cause grouping and hierarchical aggregation.

## Results

In 2023, maternal disorders accounted for an estimated 1,601.9 disability-adjusted life years (DALYs) and 25.8 deaths per 100,000 population across Sub-Saharan Africa. However, substantial subregional inequalities are evident. Central Africa bore the highest burden, with 2,592.6 DALYs [95% UI: 1,744.8–3,460.3] and 42.7 deaths [95% UI: 28.8–56.6] per 100,000—a 4.4-fold higher rate of maternal deaths compared to Southern Africa (9.4 deaths per 100,000 [95% UI: 7.1–12.2]). Western Africa recorded 1,889.6 DALYs and 30.9 deaths per 100,000, while Eastern Africa showed the lowest burden among non-Southern regions at 1,204.4 DALYs and 18.7 deaths per 100,000 (Table 1).

**Table 1:** Age-standardized DALY and death rates (per 100 000 population) for maternal disorder causes across Sub-Saharan Africa and its subregions, 2023

Causes	Sub-Saharan Africa (2023) DALYs	Sub-Saharan Africa (2023) ___Deaths	Western___DALYs	Western___Deaths	Eastern__DALYs	Eastern___Deaths	Central___DALYs	Central___Deaths	Southern___DALYs	Southern___Deaths
Maternal disorders	1601.9 [1322.1–1929.0]	25.8 [21.1–31.5]	1889.6 [1459.2–2438.2]	30.9 [23.3–40.3]	1204.4 [1024.6–1415.9]	18.7 [15.5–22.2]	2592.6 [1744.8–3460.3]	42.7 [28.8–56.6]	586.3 [447.0–743.8]	9.4 [7.1–12.2]
Maternal hemorrhage	311.0 [212.2–439.3]	5.2 [3.5–7.5]	450.0 [300.3–650.8]	7.6 [5.1–11.0]	208.3 [141.0–289.8]	3.5 [2.3–4.9]	313.7 [184.4–490.2]	5.2 [3.1–8.3]	68.4 [44.9–102.9]	1.2 [0.8–1.8]
Maternal sepsis and other maternal infections	221.0 [154.7–306.9]	3.7 [2.6–5.1]	235.9 [161.6–328.2]	3.9 [2.7–5.5]	103.9 [72.7–145.4]	1.7 [1.2–2.4]	668.7 [392.6–973.4]	11.4 [6.8–16.5]	64.5 [41.6–96.5]	1.0 [0.7–1.6]
Maternal hypertensive disorders	303.4 [231.7–386.3]	4.9 [3.7–6.3]	395.5 [273.0–532.7]	6.5 [4.5–8.8]	202.7 [151.9–247.6]	3.3 [2.4–4.0]	431.1 [274.9–611.3]	6.9 [4.4–9.8]	111.2 [80.5–150.4]	1.9 [1.3–2.6]
Maternal obstructed labor and uterine rupture	129.3 [91.3–183.5]	1.5 [0.9–2.4]	151.4 [100.9–219.1]	1.9 [1.1–3.1]	123.7 [88.2–169.8]	1.2 [0.8–1.9]	120.5 [66.0–216.9]	1.8 [0.9–3.3]	57.6 [38.5–83.1]	0.7 [0.4–1.1]
Maternal abortion and miscarriage	158.0 [98.0–246.2]	2.6 [1.6–4.0]	192.5 [116.2–297.4]	3.2 [1.9–5.0]	126.2 [80.0–199.3]	2.0 [1.3–3.3]	225.8 [132.1–374.3]	3.7 [2.1–6.1]	29.0 [18.7–44.1]	0.5 [0.3–0.7]
Ectopic pregnancy	111.4 [74.1–157.5]	1.9 [1.2–2.7]	129.7 [81.8–189.4]	2.2 [1.4–3.2]	83.8 [55.8–121.2]	1.4 [0.9–2.0]	188.9 [113.9–280.1]	3.1 [1.9–4.7]	37.6 [23.9–52.6]	0.6 [0.4–0.9]
Other direct maternal disorders	215.6 [153.6–308.3]	3.4 [2.3–5.0]	183.7 [119.3–279.4]	2.9 [1.8–4.6]	215.8 [156.7–305.6]	3.2 [2.2–4.7]	409.5 [245.5–632.0]	6.7 [4.0–10.3]	117.8 [77.2–164.0]	1.9 [1.2–2.7]
Indirect maternal deaths	105.4 [70.7–157.9]	1.8 [1.2–2.7]	97.5 [61.8–155.3]	1.7 [1.0–2.7]	103.7 [71.9–141.7]	1.7 [1.2–2.3]	172.2 [91.7–292.8]	2.9 [1.5–4.9]	60.2 [37.7–93.3]	1.0 [0.6–1.6]
Late maternal deaths	31.3 [23.7–40.4]	0.5 [0.4–0.7]	39.0 [27.6–53.0]	0.7 [0.5–0.9]	22.2 [17.0–28.3]	0.4 [0.3–0.5]	46.0 [27.8–68.5]	0.8 [0.5–1.1]	12.9 [9.3–17.5]	0.2 [0.2–0.3]
Maternal deaths aggravated by HIV/AIDS	15.6 [9.8–21.6]	0.3 [0.2–0.4]	14.3 [8.3–21.1]	0.3 [0.1–0.4]	14.2 [8.8–21.0]	0.2 [0.2–0.4]	16.2 [8.7–24.8]	0.3 [0.2–0.4]	27.1 [17.4–37.9]	0.5 [0.3–0.7]

Cause-Specific Burden: The three leading causes of maternal disorder burden differed by region. Across SSA, maternal hemorrhage was the largest contributor (311.0 DALYs per 100,000), followed by hypertensive disorders (303.4 DALYs) and maternal sepsis (221.0 DALYs). However, this pattern varied by subregion. In Central Africa, maternal sepsis dominated (668.7 DALYs [95% UI: 392.6–973.4])—the highest observed among all causes and regions—followed by hypertensive disorders (431.1 DALYs [95% UI: 274.9–611.3]). In Western Africa, maternal hemorrhage (450.0 DALYs [95% UI: 300.3–650.8]) and hypertensive disorders (395.5 DALYs) were the leading causes. Eastern Africa showed elevated rates of maternal hemorrhage (208.3 DALYs) and abortion/miscarriage complications (126.2 DALYs). Southern Africa, with overall lower rates, was notably driven by hypertensive disorders (111.2 DALYs) and ectopic pregnancy (37.6 DALYs).

Uncertainty and Data Precision: Notably, uncertainty intervals were widest in Central Africa across most causes, reflecting data sparsity in this region. For example, the 95% UI for overall maternal disorder DALYs in Central Africa ranged from 1,744.8 to 3,460.3—a 98% relative range—compared to Eastern Africa (range 24%; from 1,024.6 to 1,415.9), indicating greater model-based uncertainty in Central African estimates. This wider uncertainty must be considered when interpreting subregional comparisons for policy purposes.

Subregional Contrasts: The ratio of highest to lowest burden (Central to Southern Africa) was 4.4 for mortality and 4.4 for DALYs, indicating profound regional inequality. This geographic stratification reflects the intersection of health system capacity, infectious disease burden (particularly HIV in some settings), and maternal risk exposure across the continent.

Table 2 presents the Average Annual Percent Change (AAPC) in maternal disorder rates from 1990 to 2023. At the continental level, maternal disorder DALYs declined significantly with an AAPC of -2.8% (95% CI: -3.0 to -2.6). Eastern Africa achieved the most rapid reductions (AAPC − 4.9%; 95% CI: -5.2 to -4.7), followed by Western Africa (-1.9%) and Central Africa (-1.2%). In contrast, Southern Africa experienced a significant increase in burden over the study period (AAPC + 1.5%; 95% CI: 0.5 to 2.5). While maternal hemorrhage declined across all regions—though only non-significantly in Southern Africa—hypertensive disorders showed divergent trends: decreasing in Eastern (-5.2%) and Central (-2.6%) Africa, but rising significantly in Southern Africa (+ 1.8%). Additionally, HIV-aggravated maternal deaths showed strong declines in Eastern (-5.3%) and Central (-3.6%) Africa but increased substantially in Southern Africa (+ 3.3%).

**Table 2:** AAPC (Average Annual Percent Change) in age-standardized DALY and death rates (per 100,000), 1990–2023 (95% CI)

Causes	Sub-Saharan Africa	Western SSA	Eastern SSA	Central SSA	Southern SSA
Maternal disorders					
DALYs	-2.8 [-3.0 to -2.6]	-1.9 [-2.1 to -1.7]	-4.9 [-5.2 to -4.7]	-1.2 [-1.6 to -0.8]	1.5 [0.5 to 2.5]
Deaths	-2.8 [-3.0 to -2.6]	-1.8 [-2.0 to -1.6]	-5.1 [-5.4 to -4.8]	-1.1 [-1.5 to -0.8]	1.8 [0.8 to 2.9]
Maternal hemorrhage					
DALYs	-3.4 [-3.6 to -3.2]	-2.2 [-2.5 to -2.0]	-5.8 [-6.1 to -5.6]	-2.7 [-3.0 to -2.3]	0.1 [-0.9 to 1.1]
Deaths	-3.4 [-3.6 to -3.2]	-2.2 [-2.4 to -1.9]	-5.9 [-6.1 to -5.6]	-2.6 [-3.0 to -2.3]	0.2 [-0.8 to 1.3]
Maternal sepsis					
DALYs	-2.9 [-3.3 to -2.5]	-2.1 [-2.3 to -1.9]	-6.6 [-7.1 to -6.0]	-0.8 [-1.5 to 0.0]	1.0 [-0.3 to 2.3]
Deaths	-2.9 [-3.2 to -2.5]	-2.0 [-2.2 to -1.8]	-6.6 [-7.1 to -6.0]	-0.7 [-1.5 to 0.0]	1.1 [-0.2 to 2.4]
Hypertensive disorders					
DALYs	-2.5 [-2.7 to -2.3]	-0.7 [-1.0 to -0.3]	-5.2 [-5.4 to -4.9]	-2.6 [-2.9 to -2.3]	1.8 [1.0 to 2.7]
Deaths	-2.5 [-2.7 to -2.2]	-0.5 [-0.8 to -0.1]	-5.2 [-5.5 to -4.9]	-2.6 [-2.9 to -2.3]	2.0 [1.1 to 2.9]
Obstructed labor					
DALYs	-2.8 [-3.0 to -2.7]	-2.1 [-2.5 to -1.8]	-4.3 [-4.5 to -4.1]	-1.4 [-1.7 to -1.1]	1.0 [0.2 to 1.8]
Deaths	-3.3 [-3.5 to -3.0]	-2.6 [-3.0 to -2.1]	-5.2 [-5.5 to -4.9]	-1.0 [-1.3 to -0.7]	2.6 [1.4 to 3.8]
Abortion/Miscarriage					
DALYs	-4.2 [-4.6 to -3.9]	-2.8 [-3.1 to -2.5]	-6.7 [-7.2 to -6.1]	-1.9 [-2.1 to -1.6]	-0.7 [-1.6 to 0.2]
Deaths	-4.2 [-4.6 to -3.9]	-2.9 [-3.2 to -2.5]	-6.7 [-7.3 to -6.1]	-1.8 [-2.1 to -1.6]	-0.5 [-1.5 to 0.5]
Ectopic pregnancy					
DALYs	-1.5 [-1.6 to -1.4]	-1.8 [-1.9 to -1.6]	-3.0 [-3.1 to -2.9]	1.7 [1.2 to 2.2]	2.4 [1.8 to 3.0]
Deaths	-1.4 [-1.5 to -1.3]	-1.6 [-1.8 to -1.4]	-2.9 [-3.1 to -2.8]	1.8 [1.3 to 2.4]	2.5 [1.9 to 3.2]
Other direct disorders					
DALYs	-1.7 [-2.0 to -1.5]	-2.7 [-3.3 to -2.1]	-2.5 [-2.6 to -2.3]	1.4 [1.1 to 1.7]	2.3 [1.6 to 2.9]
Deaths	-1.8 [-2.0 to -1.5]	-2.8 [-3.5 to -2.1]	-2.7 [-2.9 to -2.5]	1.5 [1.2 to 1.8]	2.6 [1.9 to 3.3]
Indirect maternal					
DALYs	-0.9 [-1.8 to 0.1]	-0.9 [-2.2 to 0.4]	-2.2 [-2.8 to -1.6]	1.8 [0.3 to 3.3]	3.8 [2.0 to 5.5]
Deaths	-0.7 [-1.7 to 0.2]	-0.8 [-2.1 to 0.6]	-2.1 [-2.7 to -1.5]	1.9 [0.4 to 3.5]	3.9 [2.2 to 5.7]
Late maternal deaths					
DALYs	-1.1 [-1.2 to -1.0]	-0.3 [-0.4 to -0.1]	-3.2 [-3.4 to -3.0]	0.3 [0.0 to 0.5]	1.8 [1.2 to 2.4]
Deaths	-1.0 [-1.1 to -0.9]	-0.2 [-0.3 to 0.0]	-3.1 [-3.3 to -2.9]	0.3 [0.1 to 0.6]	1.9 [1.3 to 2.6]
HIV/AIDS aggravated					
DALYs	-3.1 [-3.8 to -2.5]	-1.2 [-2.0 to -0.3]	-5.3 [-5.9 to -4.7]	-3.6 [-4.1 to -3.0]	3.3 [1.6 to 5.0]
Deaths	-2.8 [-3.5 to -2.2]	-0.9 [-1.7 to 0.0]	-5.0 [-5.7 to -4.4]	-3.4 [-3.9 to -2.8]	3.6 [1.9 to 5.4]

**Fig. 1:**
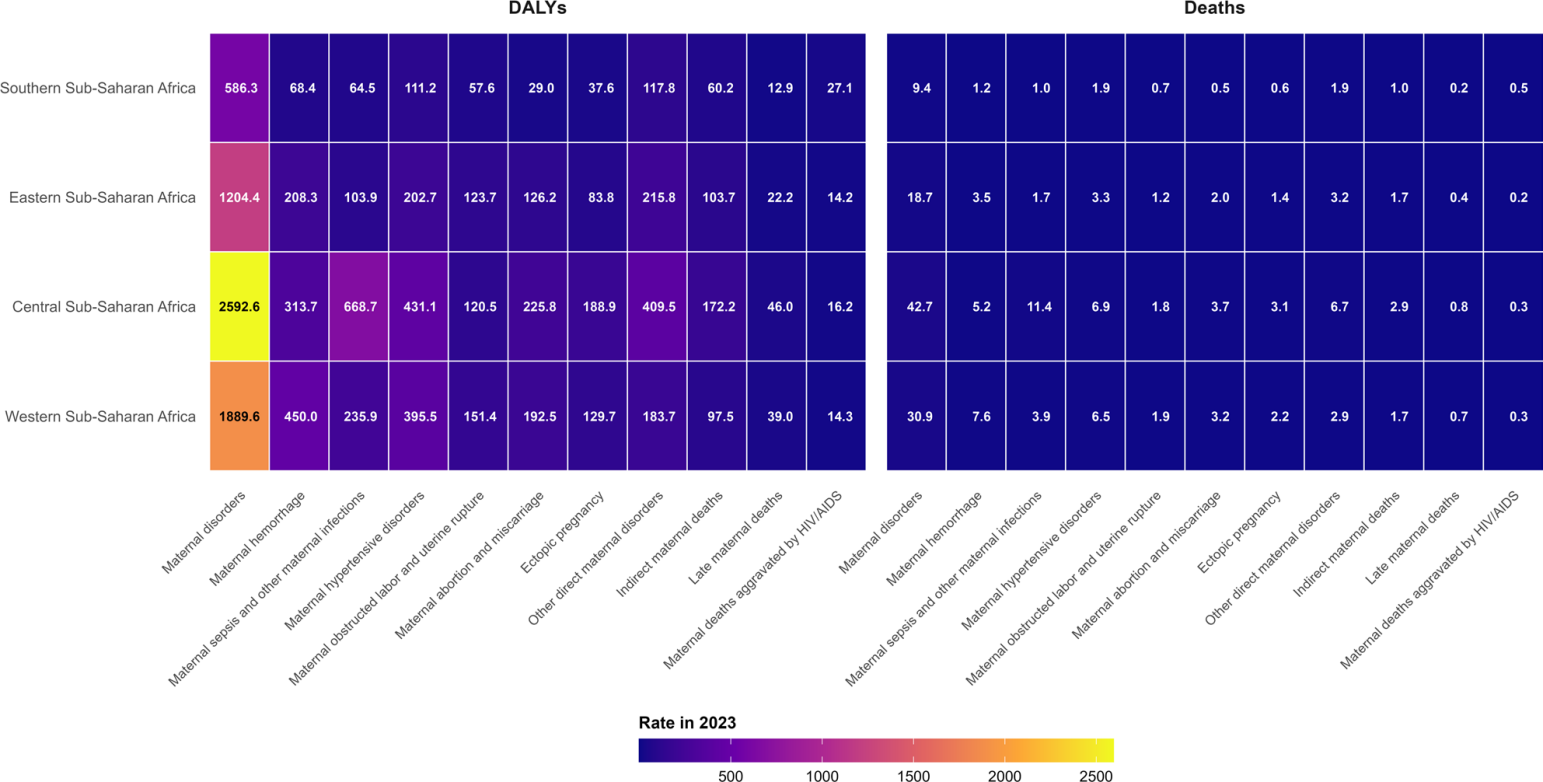
Maternal disorder DALYs and deaths across Sub-Saharan Africa subregions, 2023

Figure 1 illustrates pronounced subregional disparities in maternal disorder burden across SSA in 2023, with Central Africa experiencing the highest age-standardized DALY rate (2 592.6 per 100 000) and death rate (42.7 per 100 000), followed by Western Africa (1 889.6; 30.9), Eastern Africa (1 204.4; 18.7), and Southern Africa exhibiting the lowest rates (586.3; 9.4). Within Central Africa, maternal sepsis and other maternal infections (668.7 DALYs; 11.4 deaths) and hypertensive disorders (431.1; 6.9) stand out as key drivers, whereas in Western Africa, hemorrhage (450.0; 7.6) and hypertensive disorders (395.5; 6.5) predominate. Eastern Africa shows relatively elevated rates of obstetric hemorrhage (208.3; 3.5) and abortion and miscarriage (126.2; 2.0), while Southern Africa’s burden is notably driven by hypertensive disorders (111.2; 1.9) and ectopic pregnancy (37.6; 0.6), despite the overall lower rates. These patterns underscore the need for subregion-specific interventions targeting the leading maternal complications in each area. 

Country-level analysis reveals marked heterogeneity in maternal mortality trends across SSA (Table 3). Countries with the slowest declines cluster predominantly in Southern Africa (Zimbabwe, South Africa, Lesotho, Eswatini) and Central Africa (Somalia, Central African Republic, Democratic Republic of the Congo), with AAPC ranging from 1.1% to -0.4%. In striking contrast, Eastern African countries (Rwanda, Ethiopia, Tanzania, Malawi, Madagascar) demonstrate the fastest declines, with AAPC of -0.7% to -0.9%. This geographic stratification validates the subregional patterns observed in Table 2 and demonstrates that reported inequalities are not driven by a single high-burden country, but rather reflect distinct epidemiological trajectories linked to regional health system capacity and HIV burden.

**Table 3:** Average Annual Percent Change (AAPC) in maternal disorder death rates by country, Sub-Saharan Africa, 1990–2023 (per 100,000; 95% CI)

Rank	Country	Region	AAPC Death Rate (95% CI)
Slowest Decline (Top 10)			
1	Zimbabwe	Southern SSA	1.1 [0.3 to 2.7]
2	South Africa	Southern SSA	0.4 [-0.2 to 1.5]
3	Lesotho	Southern SSA	0.1 [-0.4 to 0.7]
4	Somalia	Central SSA	-0.1 [-0.5 to 1.2]
5	Central African Republic	Central SSA	-0.2 [-0.5 to 0.4]
6	Eswatini	Southern SSA	-0.2 [-0.6 to 0.4]
7	Gabon	Central SSA	-0.3 [-0.7 to 0.2]
8	Democratic Republic of the Congo	Central SSA	-0.3 [-0.6 to 0.0]
9	Côte d’Ivoire	Western SSA	-0.4 [-0.6 to 0.2]
10	Niger	Western SSA	-0.4 [-0.7 to 0.0]
Fastest Decline (Bottom 10)			
37	Zambia	Eastern SSA	-0.7 [-0.9 to -0.6]
38	Mauritania	Western SSA	-0.7 [-0.8 to -0.6]
39	Malawi	Eastern SSA	-0.7 [-0.8 to -0.5]
40	Madagascar	Eastern SSA	-0.7 [-0.9 to -0.5]
41	Botswana	Southern SSA	-0.8 [-0.9 to -0.5]
42	Cabo Verde	Western SSA	-0.8 [-0.9 to -0.5]
43	Equatorial Guinea	Central SSA	-0.8 [-0.9 to -0.5]
44	United Republic of Tanzania	Eastern SSA	-0.8 [-0.9 to -0.7]
45	Ethiopia	Eastern SSA	-0.9 [-0.9 to -0.8]
46	Rwanda	Eastern SSA	-0.9 [-0.9 to -0.8]

Across the 46 SSA countries, the median AAPC was − 0.8% (interquartile range: -0.9 to -0.7), with Eastern Africa showing a lower (more favorable) median of -0.9% compared to Southern Africa at 0.0% (reflecting the mix of countries with positive and negative trends). This subregional difference remained consistent after population-weighting, indicating that the observed inequalities reflect broad-based geographic variation rather than concentration in a few large nations.

**Fig. 2:**
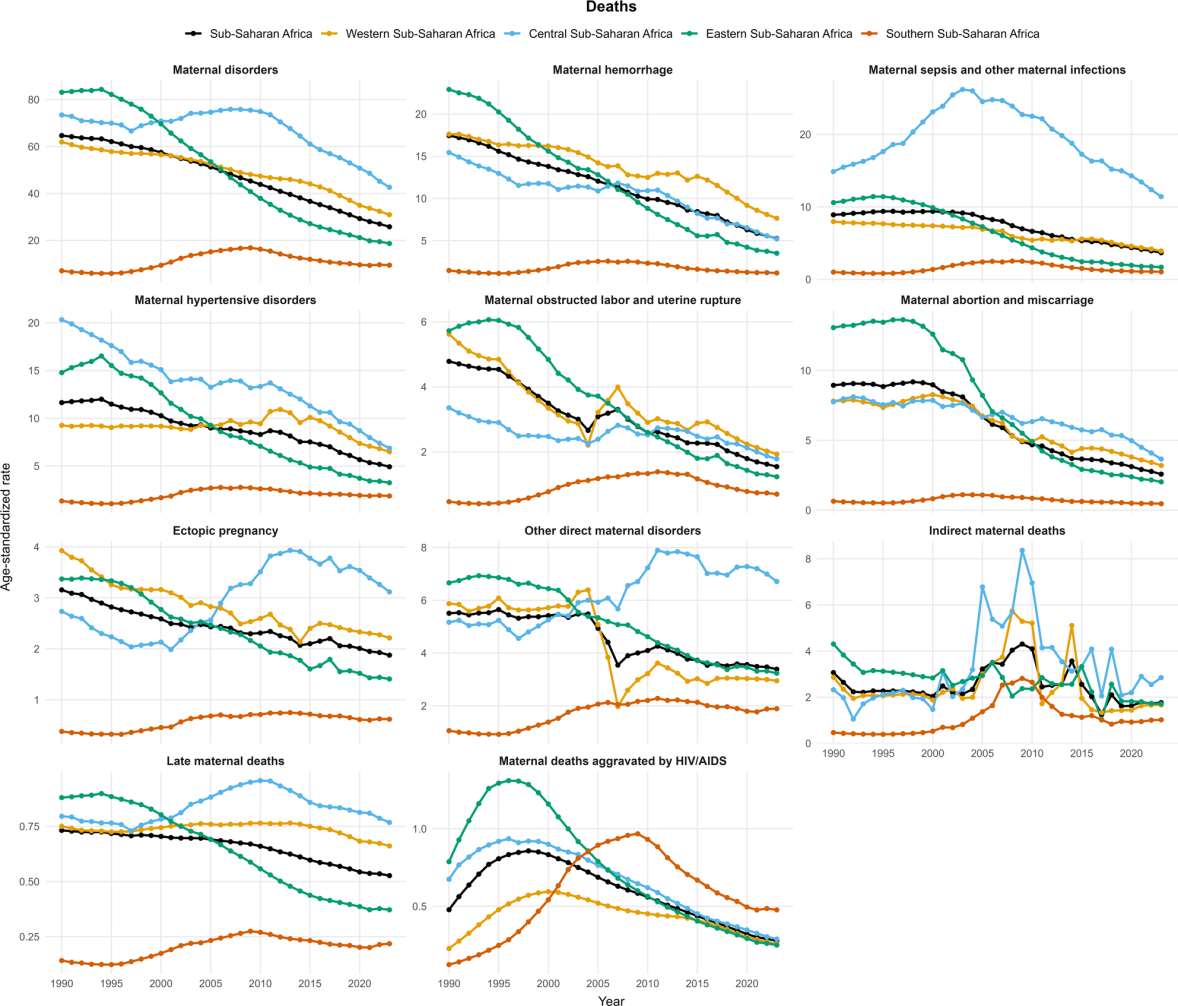
Temporal trends in maternal disorder-specific death rates across Sub-Saharan Africa subregions, 1990–2023

Figure 2 reveals distinct temporal trajectories in maternal disorder–specific mortality across the SSA subregions from 1990 to 2023. Overall, maternal death rates (upper left panel) declined steadily in Western, Eastern, and Southern Africa, whereas Central Africa exhibited slower initial decreases followed by a plateau post-2005. Maternal hemorrhage mortality fell consistently across all regions, with Southern Africa achieving the most rapid decline after 2000. Maternal sepsis mortality peaked in Central Africa around 2005 before declining, while Western and Eastern Africa showed gradual decreases, and Southern Africa maintained low and stable rates. Hypertensive disorder–related deaths declined across Eastern and Western Africa but remained elevated in Central Africa until the late 2000s and decreased only modestly thereafter; Southern Africa’s rates were the lowest and declined steadily. Obstructed labor mortality exhibited a pronounced drop in Eastern Africa around 2005, reflecting obstetric care scale-up, whereas Western and Central Africa showed more gradual reductions, and Southern Africa remained minimal. Abortion-and miscarriage–related deaths mirrored overall trends, with Eastern Africa’s sharp decline after 2000, contrasting with slower progress elsewhere. Ectopic pregnancy deaths decreased uniformly, while “other direct maternal disorders” spiked in Western and Central Africa around 2005 before converging with the downward trend. Indirect maternal deaths, largely driven by HIV/AIDS, surged in Central and Western Africa during the early 2000s Central peaking around 2007 and Western peaking around 2010 before declining following expanded antiretroviral therapy; Eastern Africa experienced a smaller peak, and Southern Africa remained low. Late maternal death rates declined modestly in Western and Eastern Africa, remained stable in Southern Africa, and increased slightly in Central Africa. Maternal deaths aggravated by HIV/AIDS showed a pronounced peak in Eastern Africa in the late 1990s, a later peak in Southern Africa around 2005, and more muted peaks in Western and Central Africa, followed by convergence toward lower rates by 2023.

**Fig. 3:**
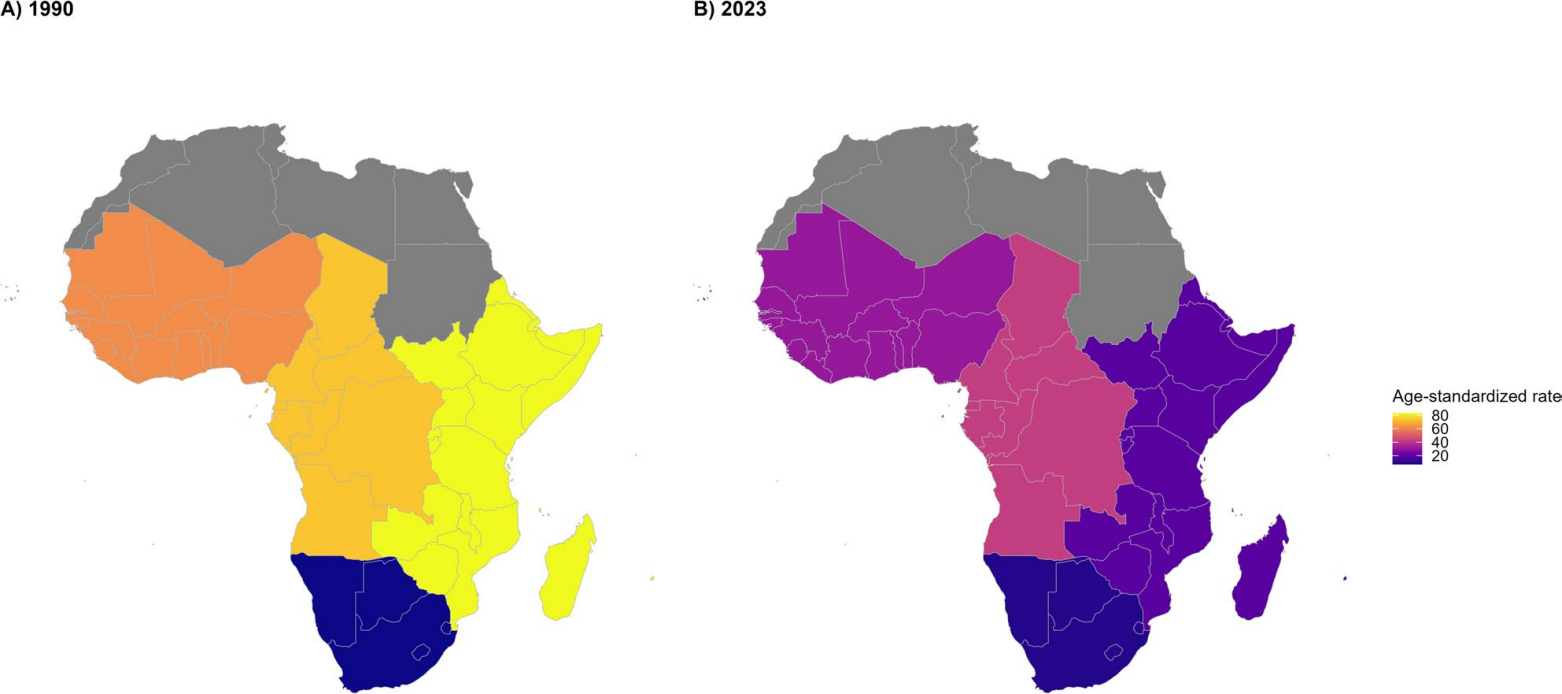
Geographic distribution of age-standardized maternal disorder death rates across Africa, 1990 and 2023

Figure 3 illustrates a marked shift in the geographical pattern of maternal disorder mortality across Africa between 1990 and 2023. In 1990, Central and Western Africa exhibited the highest age-standardized death rates (60–80 per 100 000), Eastern Africa showed intermediate rates (40–60), and Southern Africa had the lowest (< 20). By 2023, all regions will experience substantial declines, with Southern Africa maintaining the lowest rates (< 20), Central Africa, and parts of Eastern and Western Africa converging toward moderate mortality levels (20–40), reflecting the region-wide impact of maternal health interventions over three decades.

## Discussion

This study revealed substantial declines in the burden of maternal disorders across SSA between 1990 and 2023, alongside persistent and widening regional inequalities. In 2023, the overall age-standardized rate of disability-adjusted life years (DALYs) due to maternal disorders was 1,601.9 per 100,000 population, with a corresponding mortality of 25.8 deaths per 100,000. Central Africa experienced the highest burden (2,592.6 DALYs; 42.7 deaths per 100,000), followed by Western Africa (1,889.6; 30.9), Eastern Africa (1,204.4; 18.7), and Southern Africa (586.3; 9.4). The leading causes remain maternal hemorrhage, sepsis, and hypertensive disorders, with abortion/miscarriage, obstructed labor, and ectopic pregnancy also contributing substantially. From 1990 to 2023, overall DALYs and deaths declined by approximately 60%, yet reductions varied markedly by region: Eastern Africa achieved the steepest decreases (–76.6% DALYs), Central Africa saw moderate declines, Western Africa experienced slower progress, and Southern Africa recorded increases in specific causes, such as hypertensive disorders, ectopic pregnancy, and HIV-related maternal deaths. The interpretation of these trends should be situated within the broader global and regional evidence. The WHO’s 2023 maternal mortality estimates similarly highlight that while global maternal mortality ratios declined by 34% from 2000 to 2020, SSA continues to bear over two-thirds of all maternal deaths, underscoring persistent inequalities [22]. he divergent trends in Southern Africa, particularly the rise in HIV-aggravated maternal deaths and hypertensive disorders, are likely driven by the high burden in South Africa and Lesotho, where the HIV epidemic has historically intersected with maternal health outcomes, although coverage of antiretroviral therapy has improved survival in recent years.

Several factors are likely to underlie the observed regional disparities. Health system capacity varies markedly across the region, with Central and Western Africa often characterized by insufficient infrastructure, workforce shortages, and limited emergency obstetric care services [23]. Skilled birth attendance ranges from over 80% in some Eastern African countries to below 50% in parts of Central Africa. Antenatal care (ANC) coverage, particularly quality ANC, also shows wide variation and has been directly linked to maternal mortality reductions [15, 24]. Socioeconomic conditions, including poverty, educational attainment, and rural residence, further influence access to and utilization of maternal health services [25]. Higher fertility rates in Central and Western Africa increase absolute exposure to pregnancy-related risks, whereas conflict and political instability exacerbate service disruption and underreporting [23].

Global initiatives, such as the Millennium Development Goals (MDGs) and Sustainable Development Goals (SDG) 3.1, have driven much of the progress over the past three decades. The MDG 5 target of reducing the maternal mortality ratio (MMR) by three-quarters between 1990 and 2015 catalyzed substantial investments in maternal health, resulting in accelerated declines from the mid-2000s [26]. However, progress was uneven, with MDG 5 achievements concentrated in countries with stronger governance and health systems, and many Central and Western African nations missed the target [26]. While Eastern Africa shows strong declines consistent with SDG progress, Central Africa requires accelerated efforts to match these gains. SDG frameworks have emphasized universal health coverage (UHC), quality of care, and equity; however, the persisting burden in Southern Africa, driven in part by the HIV epidemic and rising hypertensive disorders, highlights gaps in translating global goals into equitable outcomes [25].

Condition-specific trends reflect the differential impact of interventions and policy reforms. Maternal hemorrhage, historically the leading cause of maternal DALYs, declined substantially across all subregions, consistent with the increased use of active management in the third stage of labor and improved blood transfusion services [27]. Sepsis saw moderate reductions, likely due to expanded antibiotic prophylaxis and improved infection control, yet remains problematic in settings with limited sterile supplies and delayed referral systems [1]. Hypertensive disorders improved most in Eastern and Central Africa, where expanded magnesium sulfate availability and protocolized management have been prioritized. Southern Africa’s stagnation may reflect rising baseline hypertension prevalence, linkage to HIV treatment side effects, and gaps in early detection during ANC [1]. Obstructed labor and ectopic pregnancy exhibited slower declines; prevention of obstructed labor hinges on antenatal identification of high-risk pregnancies and timely access to cesarean delivery, which remains constrained in rural and underresourced areas [28].

The social determinants play a critical role in this process. Female education is inversely associated with maternal disorder burden through multiple pathways, including increased health literacy, empowerment to seek care, and delayed age at first pregnancy [25]. Poverty constrains access to nutritious diets, skilled attendance, and transportation to healthcare facilities [15]. Gender inequalities limit women’s decision-making and control over their reproductive health choices [25]. Access to comprehensive reproductive health services, including family planning and safe abortion, directly reduces abortion-related morbidity. Restrictive legal environments and stigma in certain countries perpetuate high DALYs from abortion and miscarriage [28].

Comparisons with prior studies underscore both the progress and persistent gaps. A GBD 2021 analysis reported a 58% decline in maternal disorder DALYs globally from 1990 to 2021, with sub-Saharan Africa showing a slightly slower 52% reduction [1]. Our findings extend these trends to 2023 and highlight sharper improvements in Eastern Africa. Evidence from Demographic and Health Surveys (DHS) (2015–2021) indicates that quality ANC, measured by the receipt of essential components such as blood pressure measurement and tetanus immunization, remains suboptimal in Central and Western Africa, correlating with higher sepsis and hypertensive disorder burden [24]. Studies between 2018 and 2024 have documented the efficacy of midwife-led birthing centers and community health worker programs in Ethiopia and Rwanda in reducing hemorrhage and sepsis, illustrating scalable models for other regions [29].

## Limitations and conclusion

This study has some limitations including residual uncertainty in GBD estimates due to incomplete vital registration systems, potential underreporting of maternal deaths (particularly indirect causes), and variability in input data quality across countries. The ecological nature of subregional analyses precludes causal inferences at the national and subnational levels. Finally, rapid changes in conflict zones and health system disruptions due to COVID-19 may not be fully captured in the 2023 estimates. Despite a remarkable 60% reduction in the burden of maternal disorders across SSA from 1990 to 2023, significant regional inequalities persist. Central and Western Africa remain disproportionately affected, whereas Southern Africa faces emerging challenges from hypertensive disorders and HIV-related maternal deaths. To achieve SDG 3.1 equitably, policies must strengthen health system capacity, ensure universal access to high-quality antenatal and obstetric care, expand reproductive health services, and address social determinants through multi-sectoral interventions. Global initiatives must be matched by sustained domestic investment and targeted strategies in lagging regions to close this gap and safeguard maternal health gains.

This study relies on GBD 2023 modeled estimates, which utilize available data and covariate-based predictive modeling (CODEm) to fill gaps where primary data are sparse. Consequently, estimates for subregions with limited vital registration systems, particularly Central Africa, are subject to wider uncertainty intervals and rely heavily on borrowing strength from covariate associations. We have presented 95% uncertainty intervals throughout to reflect this modeled precision. Additionally, subregional aggregations may mask heterogeneity at the national level; for example, trends in Southern Africa are heavily influenced by South Africa’s population size and epidemiological profile.

## Supplementary Information

Supplementary Material 1.

## Data Availability

The datasets analyzed during the current study are publicly available from the Global Burden of Disease 2023 database and can be accessed through the Global Health Data Exchange platform.
