# Supplementary material for: Regional inequalities and temporal trends in maternal disorders across sub-Saharan Africa, 1990–2023: a systematic analysis of the global burden of disease 2023 data
Source: Arch Public Health. 2026 Jun 4;84:162. doi: 10.1186/s13690-026-01979-z (PMC13383567; doi:10.1186/s13690-026-01979-z)
Supplement: Supplementary file 1 — Supplementary Material 1. [file 13690_2026_1979_MOESM1_ESM.docx]

| **Supplementary Table S1. Country-level age-standardized maternal disorder death and DALY rates per 100,000 population among females in Sub-Saharan Africa, 2023 (95% uncertainty intervals).** | | |
| --- | --- | --- |
|  |  |  |
| **Country** | **Death rate (per 100,000)** | **DALY rate (per 100,000)** |
| Angola | -0.7 [-0.8–-0.4] | -0.7 [-0.8–-0.4] |
| Benin | -0.6 [-0.8–-0.3] | -0.6 [-0.8–-0.3] |
| Botswana | -0.8 [-0.9–-0.5] | -0.7 [-0.9–-0.5] |
| Burkina Faso | -0.6 [-0.8–-0.3] | -0.6 [-0.8–-0.4] |
| Burundi | -0.7 [-0.8–-0.4] | -0.7 [-0.8–-0.4] |
| Cabo Verde | -0.8 [-0.9–-0.5] | -0.8 [-0.9–-0.5] |
| Cameroon | -0.5 [-0.7–-0.2] | -0.5 [-0.7–-0.2] |
| Central African Republic | -0.2 [-0.5–0.4] | -0.2 [-0.5–0.4] |
| Chad | -0.4 [-0.6–-0.1] | -0.4 [-0.6–-0.2] |
| Comoros | -0.7 [-0.9–-0.4] | -0.7 [-0.8–-0.5] |
| Congo | -0.6 [-0.8–-0.3] | -0.6 [-0.8–-0.3] |
| CÃ´te d'Ivoire | -0.4 [-0.6–0.2] | -0.4 [-0.6–0.1] |
| Democratic Republic of the Congo | -0.3 [-0.6–0.0] | -0.3 [-0.6–0.0] |
| Djibouti | -0.5 [-0.7–0.0] | -0.5 [-0.7–-0.0] |
| Equatorial Guinea | -0.8 [-0.9–-0.5] | -0.8 [-0.9–-0.5] |
| Eritrea | -0.7 [-0.8–-0.5] | -0.7 [-0.8–-0.5] |
| Eswatini | -0.2 [-0.6–0.4] | -0.3 [-0.6–0.3] |
| Ethiopia | -0.9 [-0.9–-0.8] | -0.9 [-0.9–-0.8] |
| Gabon | -0.3 [-0.7–0.2] | -0.3 [-0.7–0.2] |
| Gambia | -0.5 [-0.8–-0.1] | -0.5 [-0.8–-0.1] |
| Ghana | -0.6 [-0.8–-0.3] | -0.6 [-0.8–-0.3] |
| Guinea | -0.6 [-0.7–-0.3] | -0.6 [-0.7–-0.4] |
| Guinea-Bissau | -0.6 [-0.8–-0.2] | -0.6 [-0.8–-0.2] |
| Kenya | -0.6 [-0.8–-0.4] | -0.6 [-0.8–-0.4] |
| Lesotho | 0.1 [-0.4–0.7] | -0.0 [-0.4–0.6] |
| Liberia | -0.4 [-0.7–-0.1] | -0.4 [-0.7–-0.1] |
| Madagascar | -0.7 [-0.9–-0.5] | -0.7 [-0.8–-0.5] |
| Malawi | -0.7 [-0.8–-0.5] | -0.7 [-0.8–-0.6] |
| Mali | -0.6 [-0.8–-0.4] | -0.6 [-0.7–-0.4] |
| Mauritania | -0.7 [-0.8–-0.6] | -0.7 [-0.8–-0.6] |
| Mozambique | -0.7 [-0.8–-0.5] | -0.7 [-0.8–-0.5] |
| Namibia | -0.7 [-0.8–-0.3] | -0.7 [-0.8–-0.4] |
| Niger | -0.4 [-0.7–0.0] | -0.4 [-0.6–0.0] |
| Nigeria | -0.5 [-0.7–-0.1] | -0.5 [-0.7–-0.1] |
| Rwanda | -0.9 [-0.9–-0.8] | -0.9 [-0.9–-0.8] |
| Sao Tome and Principe | -0.7 [-0.8–-0.3] | -0.7 [-0.8–-0.4] |
| Senegal | -0.7 [-0.8–-0.6] | -0.7 [-0.8–-0.6] |
| Sierra Leone | -0.5 [-0.7–-0.3] | -0.5 [-0.7–-0.3] |
| Somalia | -0.1 [-0.5–1.2] | -0.1 [-0.5–1.0] |
| South Africa | 0.4 [-0.2–1.5] | 0.3 [-0.2–1.2] |
| South Sudan | -0.5 [-0.7–0.0] | -0.5 [-0.7–0.0] |
| Togo | -0.5 [-0.7–-0.0] | -0.5 [-0.7–-0.1] |
| Uganda | -0.5 [-0.8–-0.1] | -0.5 [-0.7–-0.2] |
| United Republic of Tanzania | -0.8 [-0.9–-0.7] | -0.8 [-0.9–-0.7] |
| Zambia | -0.7 [-0.9–-0.6] | -0.7 [-0.8–-0.6] |
| Zimbabwe | 1.1 [0.3–2.7] | 0.9 [0.2–2.2] |
